# Injection of Alcohol in Uterine Carcinoma

**Published:** 1892-08

**Authors:** 


INJECTION OF ALCOHOL IN UTERINE CARCINOMA.—Dr. H. Schultz, in *Centralblatt fur Gynœcologie*, makes a preliminary report of the treatment of uterine carcinoma with injections of absolute alcohol. In one case of disease of the cervix there was a complete cure. In nine other cases, which are still under observation, there is marked improvement. The injections are made deep into the affected tissues.—*Notes on New Remedies.*

